# Attenuation of Acute Renal Injury After the Post-resuscitation Administration of Doxycycline in Surviving Newborn Piglets With Severe Hypoxia-Reoxygenation

**DOI:** 10.3389/fped.2019.00075

**Published:** 2019-03-22

**Authors:** Tze-Fun Lee, Min Lu, Matteo P. Pasquin, Georg M. Schmölzer, Po-Yin Cheung

**Affiliations:** ^1^Department of Pediatrics, University of Alberta, Edmonton, AB, Canada; ^2^Centre for the Studies of Asphyxia and Resuscitation, University of Alberta, Edmonton, AB, Canada; ^3^Department of Biotechnology and Biosciences, Università degli Studi di Milano-Bicocca, Milan, Italy; ^4^Department of Pharmacology, University of Alberta, Edmonton, AB, Canada

**Keywords:** renal failure, asphyxia, MMP, doxycycline, newborn

## Abstract

**Background:** Asphyxiated neonates often have myocardial dysfunction and renal insufficiency. Previously we demonstrated that doxycycline improved cardio-renal function through matrix metalloproteinase (MMP)-2 inhibition in an acute swine model of neonatal hypoxia-reoxygenation. The prolonged cardio-renal protective effects of doxycycline in neonates still remained unknown. We therefore hypothesized that the protective effects of doxycycline persisted in surviving subjects.

**Methods:** Newborn piglets were instrumented and subjected to 1 h of hypoxia followed by reoxygenation with 21–25% oxygen and observed for 4 days. Intravenous doxycycline (30 mg/kg) or normal saline (1 mL, saline-control group) was given at 5 min of reoxygenation (*n* = 8/group) in a randomized, blinded fashion. Sham-operated piglets (*n* = 5) received no hypoxia-reoxygenation. At 96 h after reoxygenation, the left ventricular function was assessed by Millar® catheter. Renal injury was investigated by measuring plasma creatinine, urinary N-acetyl-D-glucosaminidase activity, renal tissue lactate and MMP-2 activity.

**Results:** Both hypoxia-reoxygenation groups had similar hypoxic stress with severe lactate acidosis, and hemodynamic recovery. Doxycycline-treated piglets had higher urine output with lower urine N-acetyl-D-glucosaminidase, plasma creatinine, and renal MMP-2 activity (vs. saline-controls; all *p* < 0.05). These markers were all negatively correlated with urine output.

**Conclusions:** In newborn piglets surviving hypoxia-reoxygenation, we observed a weak but significant and persistent attenuation of renal injury and improved recovery with the post-resuscitation administration of doxycycline.

## Introduction

Despite recent advances in obstetrical care and newborn resuscitation, asphyxia remains a major cause of neonatal mortality and morbidity. Annually, of the estimated 4 million neonatal deaths, approximately 23% are a result of asphyxia (1–3). Asphyxiated neonates commonly have multi-organ dysfunction and/or failure (4). Because of preferential perfusion of the vital organs such as the heart and brain during hypoxia, kidney is among the first organ injured by an hypoxic-ischemic insult due to regional vasoconstriction (5–7). Indeed, acute kidney injury (AKI) has been reported to be presented in 30–70% of asphyxiated neonates (4–6). AKI has been shown to be associated with neonatal asphyxia, the outcome and its subsequent neurodevelopment in early childhood (5). Perlman and Tack previously suggested that oliguria in the perinatal period was a sensitive indicator of infants at risk for long-term neurologic deficits (8).

In addition to the hypoxic and ischemic injury to kidneys, the production of oxygen free radical species and the resultant oxidative stress contribute to further renal dysfunction during reoxygenation. It has been shown that increased oxidative stress can lead to the activation of matrix metalloproteinases (MMP), a family of zinc dependent endopeptidases with a variety of intracellular and extracellular proteolytic substrates (9). They are involved in the remodeling of the extracellular matrix in tissue during various physiological and pathologic conditions. Previous findings suggest that MMPs, commonly MMP-2 and MMP-9, play a crucial role in the pathogenesis of acute ischemia-reperfusion (I-R) injury of the kidney (10, 11). Further, it has been shown that the degree of acute tubular injury, necrosis, apoptosis and renal dysfunction was markedly less in the MMP-2 deficient transgenic mice compared to that seen in the wild type mice (12).

Accordingly, inhibition of MMPs has been suggested to be used as a clinically useful target for minimizing AKI after hypoxia-reoxygenation (H-R). Doxycycline (DOX) is an antibiotic that is approved for clinical use in neonates and other patient populations (13). DOX has selective inhibitory effects on MMP-2 and MMP-9, which is independent of its antimicrobial properties (14, 15). The renal protective effects of DOX have been shown in adult rat models of I-R renal injury (16, 17). Its protective effect in neonates against AKI induced by H-R or I-R is largely unknown. Previously, we also demonstrated that post-resuscitation administration of DOX attenuated AKI associated with the inhibition of MMP-2 activity in newborn piglets with severe H-R (18). Despite providing functional and biochemical data for the beneficial effects of DOX in neonatal H-R, the short time course (4 h after reoxygenation) precludes us from understanding if the renal protection of DOX will persist beyond the acute stage.

Using a surviving model of neonatal H-R, we aimed to examine whether the renal protective effects of intravenous DOX persisted for days after the newborn piglets recovered from H-R, which is important in the translation of findings to clinical trials. We also examined the mechanism of action of the renal protective actions of DOX in neonatal H-R. We hypothesized that post-resuscitation administration of DOX in newborn piglets with severe H-R would improve renal function with alleviated renal injury and inhibition of MMP-2 activity.

## Methods

Twenty-one newborn mixed breed piglets (1–4 days of age, weighing 1.7–2.4 kg) were obtained on the day of experimentation from the University Swine Research Technology Center. All experiments were conducted in strict accordance with the guidelines and approval of the Animal Care and Use Committee (Health Sciences), University of Alberta. The ARRIVE guidelines were also followed (19).

### Animal Preparation

Piglets were anesthetized with inhaled isoflurane (1–5%) throughout the surgical procedure. During the experiment anesthesia was maintained with intravenous propofol (5–10 mg/kg/h) and morphine (0.1 mg/kg/h). Additional doses of propofol (1–2 mg/kg) and morphine (0.05–0.1 mg/kg) were also given as needed. The animals were orally intubated with an endotracheal tube (#3.5 mm, Mallinckrodt™, Covidien IIc, Mansfield, MA). Piglets were mechanically ventilated at a rate of 16–20 breaths/min at pressures of 20/5 cmH_2_O. Oxygen saturation was kept within 90–100%, hydration was maintained with an intravenous infusion of 10% dextrose solution at 10 mL/kg/h. The piglet's body temperature was maintained at 38.5–39.5°C using an overhead warmer and a water heating pad.

### Surgical Procedures and Hemodynamic Parameters Monitoring

Via a neck incision, a 5F double-lumen umbilical catheter (Argyle®, Klein-Baker Medical, San Antonio, TX) was subcutaneously tunneled and inserted into the right external jugular vein for the administration of medications and fluids. Another single-lumen umbilical catheter (5F) was subcutaneously tunneled and inserted into the right common carotid artery for systemic blood pressure monitoring and blood sampling. At the end of the surgical procedure, the neck incision was closed with sutures. On the 4th day of the experiment, piglet was anesthetized and a Millar® catheter (MPVS Ultra®, ADInstruments, Houston, TX) was inserted into the left ventricle via the left common carotid artery for continuous measurement of left ventricular contractile function.

Piglets recovered from surgical instrumentation until baseline hemodynamic parameters were stable (defined as ± <10% changes). Ventilator rate was adjusted to maintain normocapnia (paCO_2_ 35–45 mmHg). Systemic mean arterial pressure and heart rate were continuously measured and recorded throughout the experiment with a Hewlett Packard 78833B monitor (Hewlett Packard Co., Palo Alto, CA).

### Experimental Protocol (Figure 1)

Piglets were block-randomized into two treatment groups (*n* = 8/group) and subjected to an 1-h period of normocapnic hypoxemia (FiO_2_ of 0.11–0.15) followed by a 4-h period of normoxic reoxygenation (FiO_2_ of 0.21–0.25). Five minutes into reoxygenation piglets received an intravenous bolus of either 1 mL normal saline (saline-control group) or 30 mg/kg DOX (DOX group), an optimal dose based on our previous study (18), *blindly*. Five sham-operated piglets underwent surgery with no hypoxia and reoxygenation at a FiO_2_ of 0.21–0.25. After fully recovered from H-R with satisfactory respiratory status, the piglets were extubated to spontaneous breathing in room air and recovered for 4 days. After hemodynamic measurements with Millar® catheter on day 4th, the animal was euthanized with an intravenous overdose of phenobarbital (100 mg/kg) and tissues were snap frozen in liquid nitrogen and stored in −80°C until subsequent analysis.

**Figure 1:**
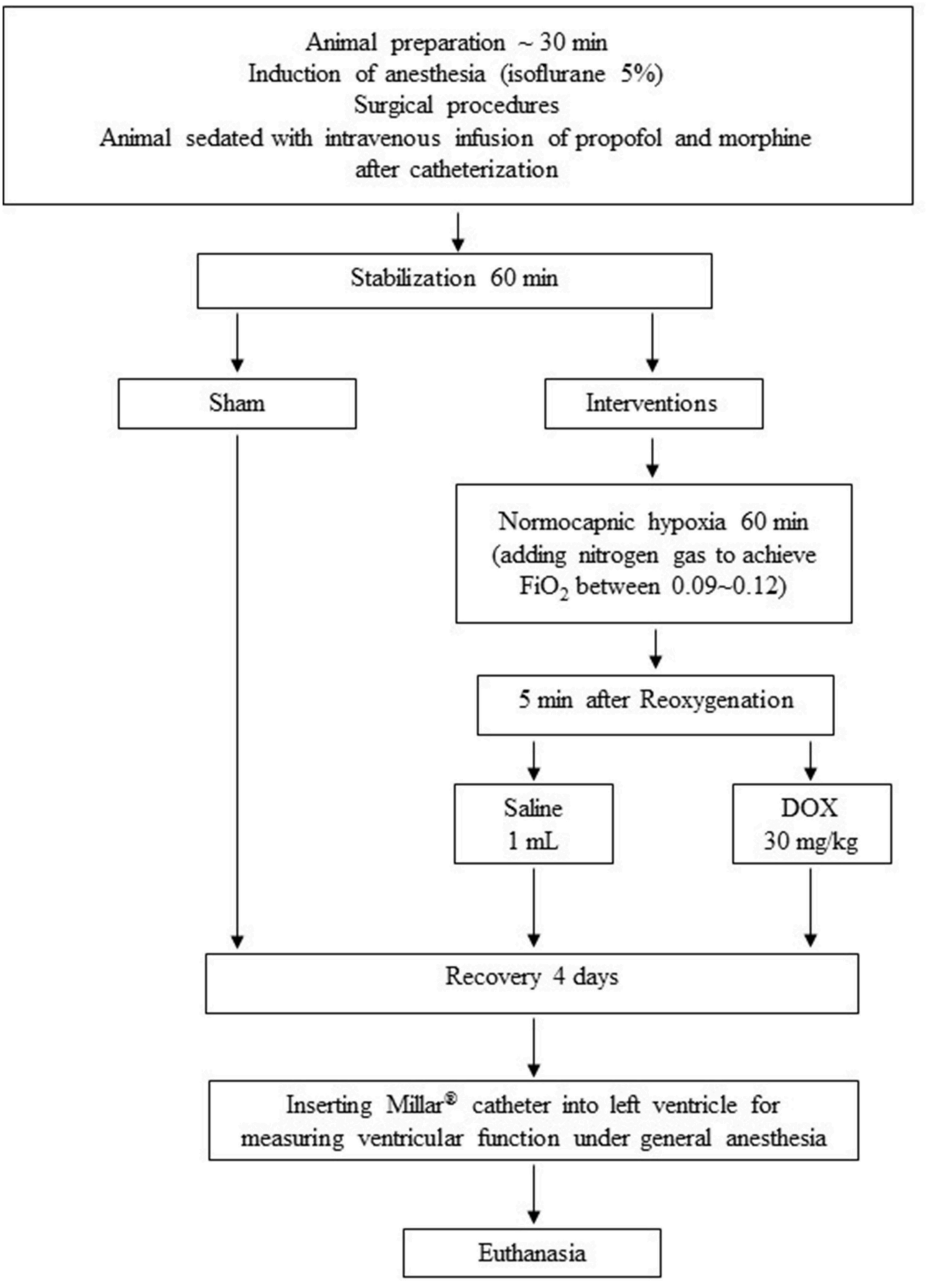
Experimental protocol.

### Postoperative Care

Postoperatively, the piglet was housed in individual kennel to which the animal was secured by a tether-swivel system (Lomir Biomedical Inc., Notre-Dame-de-l'Île-Perrot, QC). The ambient temperature was maintained at 28 ± 1°C and lighting was established with a 12-h light/dark cycle. Piglets' behaviors, including lethargy, lack of interest in surroundings, abnormal cry, vomiting, and temperature were monitored every 4 h per day (from 0800 to 2400 h). Post-operative pain and discomfort were minimized by intravenous buprenorphine (0.01 mg/kg) Q12h and oral acetaminophen (15 mg/kg) Q8h, Cefazolin (10 mg/kg) Q8h were given intravenously to prevent sepsis. Hibitane (Wyeth Animal Health, Guelph, ON) was also applied to the incision area to prevent potential wound infection. At 1200 h every day, the piglets were weighed, and nutrition solution infusion rates were calculated according to the body weight. All piglets were not catheterized and had spontaneous urination. A specially designed pan which was placed underneath the housing kennel was used to collect uncontaminated urine. The urine was removed, and its volume was measured daily at 1200 h. Blood pressure and heart rate were recorded via arterial catheter. Rate-pressure product, a parameter of cardiac performance in intact animals, was calculated as heart rate x mean arterial pressure. Plasma and urine samples were also collected and stored in −80°C until subsequent analysis.

Intravenous solution was given through a controlled pressure-sensitive infusion pump (IVAC Signature Gold Infusion Pump; ALARIS Medical Systems, San Diego, CA). Immediately after putting the piglet in the kennel (day 0), all piglets were given 10% dextrose solution at 10 mL/kg/h. Parenteral nutrition was initiated on day 1 and gradually increased to 10 mL/kg/h on day 2. The parenteral nutrition was estimated from daily nutritional requirements for sow-fed piglets and has previously demonstrated normal growth and body composition (20). The targeted energy intake was 270 kcal/kg/d, with amino acids providing 27%, carbohydrate 37%, and lipid 36% of energy.

### Preparation of Kidney Tissue and Determination of Kidney Injury Markers

At the termination of experiment, right kidneys were immediately removed *en bloc* and snap-frozen in liquid nitrogen for biochemical analyses. The whole kidney was homogenized. Renal tissue lactate levels were determined by a nicotinamide adenine dinucleotide enzyme coupled colorimetric assay as previously described (18). Gelatinolytic activities of MMP-2 and MMP-9 were quantified using gelatin zymography as previously described (21). Tissue protein content was quantified using a bicinchoninic acid assay kit (Sigma-Aldrich Canada Ltd., Oakville, ON).

Both plasma and urine creatinine (Cr) levels were measured using a commercially available QuantiChrom assay kit (DICT-500; Bioassay Systems, Hayward, CA). N-Acetyl-D-glucosaminidase (NAG) activity was measured in the urine sample by a commercially available colorimetric assay kit (no. 875406; Roche, Indianapolis, IN) and normalized to urine Cr level.

### Statistical Analysis

Results are presented as the mean ± SEM. Based on our previous experience and a predicted 50% improvement in renal function, we required 8 animals in each H-R group. Hemodynamic and biochemical variables were compared using one-way and two-way repeated measures analysis of variance as appropriate, followed by Tukey *post-hoc* testing (SigmaPlot v13; Systat Software Inc., San Jose, CA). Correlations were determined using the Pearson Product Moment test. Significance was defined as *p* < 0.05.

## Results

**Figure 2:**
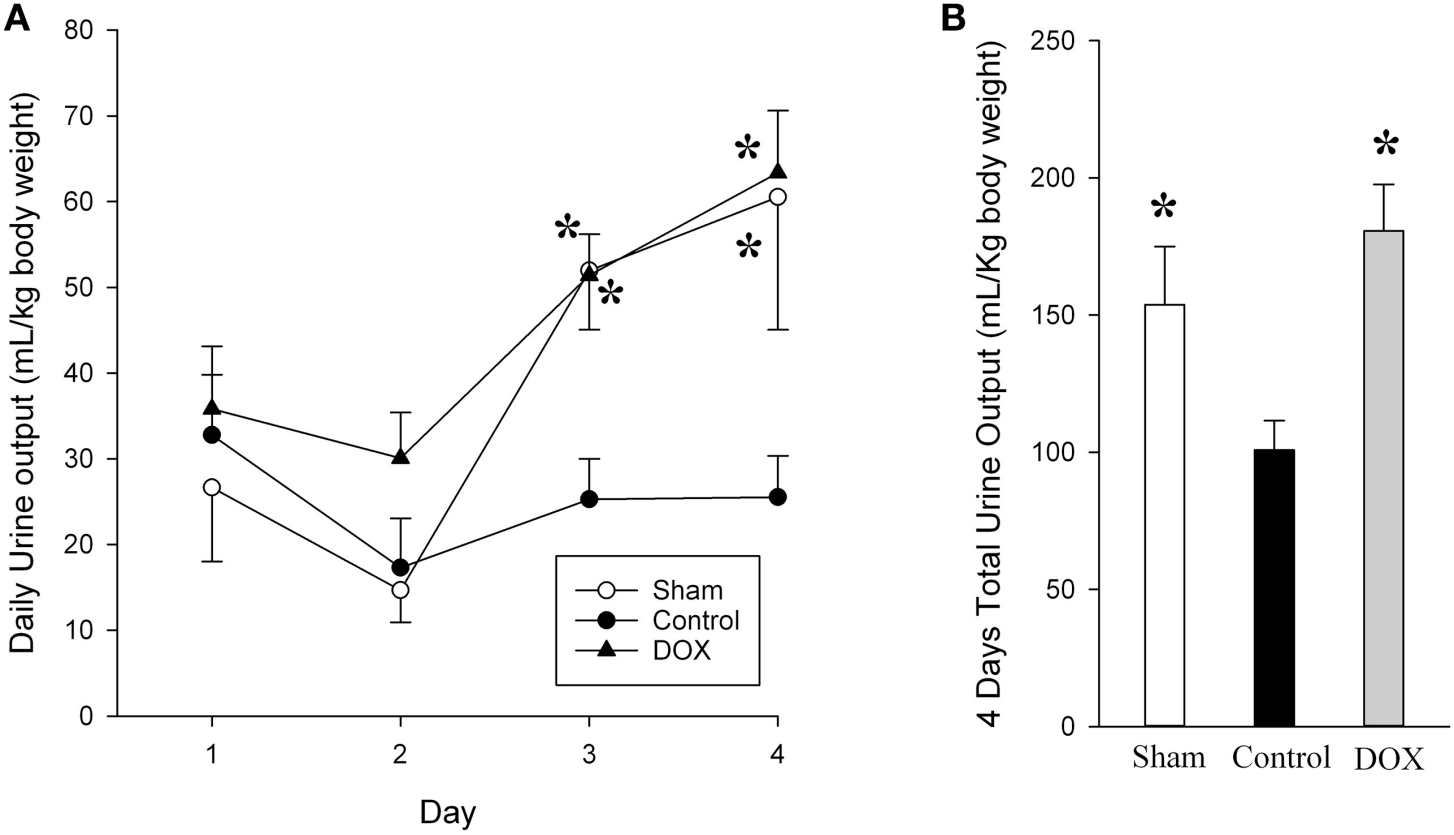
Daily **(A)** and total urine output **(B)** for sham-operated, saline-control and DOX groups after recovery. All values represent mean ± SEM. ^*^p < 0.05 vs. saline-controls at current time point (two-way repeated measures ANOVA or one-way ANOVA).

Twenty-one piglets were studied, both age and weight are similar between groups (age: 2.1 ± 0.5, 2.0 ± 0.2, and 2.6 ± 0.3 days; weight: 2.0 ± 0.1, 1.9 ± 0.1, and 2.1 ± 0.1 kg; for sham-operated, saline-control and DOX groups, respectively). There were no significant differences in baseline hemodynamic and biochemical parameters among groups (Table 1 and Figure 2).

**Table 1:** Changes in arterial blood gases during hypoxia and recovery.

	**Normoxic baseline**	**End of Hypoxia**	**Extubation**	**Recovery**			
				**Day 1**	**Day 2**	**Day 3**	**Day 4**
**pH**							
Sham-operated	7.50 ± 0.03	7.44 ± 0.05	7.45 ± 0.04	7.42 ± 0.03	7.41 ± 0.01	7.40 ± 0.02	7.39 ± 0.02
Saline-control	7.44 ± 0.02	7.04 ± 0.05^*^^†^	7.48 ± 0.03	7.46 ± 0.05	7.39 ± 0.02	7.37 ± 0.02	7.38 ± 0.02
DOX	7.43 ± 0.02	7.07 ± 0.05^*^^†^	7.48 ± 0.02	7.50 ± 0.03	7.38 ± 0.02	7.37 ± 0.02	7.42 ± 0.02
**PaO**_**2**_ **(mmHg)**							
Sham-operated	66 ± 6	68 ± 6	59 ± 6	70 ± 4	63 ± 5	72 ± 3	80 ± 10
Saline-control	62 ± 2	20 ± 3^*^^†^	61 ± 2	62 ± 2	67 ± 3	68 ± 4	67 ± 2
DOX	61 ± 6	21 ± 2^*^^†^	55 ± 8	57 ± 7	60 ± 8	69 ± 3	75 ± 5
**PaCO**_**2**_ **(mmHg)**							
Sham-operated	33 ± 2	35 ± 2	38 ± 2	34 ± 1	32 ± 1	31 ± 1	37 ± 4
Saline-control	39 ± 3	46 ± 4	33 ± 2	33 ± 2	32 ± 2	32 ± 1	35 ± 1
DOX	39 ± 2	44 ± 3	34 ± 2	31 ± 1	32 ± 2	32 ± 2	35 ± 2
$\text{HC}{\text{O}}_{3}^{-}\left(\text{mmol}/\text{L}\right)$							
Sham-operated	25.6 ± 1.6	27.8 ± 1.3	26.0 ± 1.0	22.0 ± 1.5	20.6 ± 0.5	18.9 ± 1.1	21.3 ± 1.3
Saline-control	28.1 ± 0.9	12.7 ± 1.3^*^^†^	25.0 ± 1.8	23.6 ± 1.6	18.9 ± 0.5	18.6 ± 0.9	21.2 ± 0.5
DOX	26.7 ± 0.9	13.6 ± 1.2^*^^†^	25.8 ± 0.8	24.4 ± 0.9	20.2 ± 0.9	18.9 ± 1.8	22.5 ± 1.1
**LACTATE (mmol/L)**							
Sham-operated	2.73 ± 0.5	1.53 ± 0.2	1.10 ± 0.16^†^	2.47 ± 0.3	1.09 ± 0.3	1.58 ± 0.7	1.08 ± 0.2
Saline-control	2.24 ± 0.2	14.0 ± 1.0^*^^†^	2.17 ± 0.84	3.24 ± 1.5	1.50 ± 0.5	1.13 ± 0.3	1.05 ± 0.3
DOX	2.43 ± 0.2	13.4 ± 0.8^*^^†^	1.83 ± 0.25	1.82 ± 0.3	0.96 ± 0.2	1.13 ± 0.2	0.59 ± 0.1

† p < 0.05 vs. normoxic baseline;

* *p < 0.05 vs. saline-controls at concurrent time point (2-way repeated measures ANOVA)*.

### Hemodynamic Parameters

As shown in Table 1, PaO_2_, pH and plasma bicarbonate levels were significantly lower and plasma lactate significantly higher in piglets subjected to H-R compared with sham-operated piglets at the end of hypoxia. However, there was no difference between the two H-R groups regarding the degree of hypoxic stress as indicated by similar paO_2_, metabolic acidosis and hyperlactatemia. Heart rates of both H-R groups also increased markedly at the end of hypoxia, resulting in higher rate-pressure product as compared to the sham-operated group (Table 2).

**Table 2:** Temporal changes in heart rate, mean arterial pressure (MAP) and rate-pressure-product (RPP) during hypoxia and recovery.

	**Normoxic Baseline**	**End of Hypoxia**	**Extubation**	**Recovery**			
				**Day 1**	**Day 2**	**Day 3**	**Day 4**
**HEART RATE (bpm)**							
Sham-operated	162 ± 8	163 ± 7	188 ± 6	192 ± 12	192 ± 4	209 ± 13	218 ± 13
Saline-control	175 ± 8	239 ± 26^*^^†^	200 ± 13	214 ± 9^†^	217 ± 14†	225 ± 16^†^	201 ± 6^†^
DOX	172 ± 7	236 ± 15^*^^†^	201 ± 11	207 ± 8^†^	199 ± 7	226 ± 6^†^	208 ± 10†
**MAP (mmHg)**							
Sham-operated	60 ± 6	56 ± 4	51 ± 4	59 ± 7	58 ± 5	66 ± 7	63 ± 4
Saline-control	63 ± 2	71 ± 7	53 ± 3	54 ± 2	57 ± 2	66 ± 4	57 ± 3
DOX	67 ± 4	60 ± 7	56 ± 2	48 ± 2^†^	50 ± 2^†^	57 ± 3	62 ± 2
**RPP**							
Sham-operated	9,559 ± 742	9,131 ± 597	9,644 ± 708	11,061 ± 2872	11,511 ± 995	15,021 ± 2,194	14,157 ± 1,757
Saline-control	10,962 ± 496	17783 ± 1,485^*^^†^	10,210 ± 785	11,647 ± 707	12,341 ± 850	14,964 ± 1816	11,552 ± 726
DOX	11,242 ± 987	16,221 ± 1,843^*^^†^	11,417 ± 884	9,945 ± 643	9,972 ± 687	12,838 ± 810	12,917 ± 899

† p < 0.05 vs. normoxic baseline;

* *p < 0.05 vs. saline-controls at concurrent time point (2-way repeated measures ANOVA)*.

Three piglets (one from saline-control and two from DOX) were euthanized earlier during the 4-day observation period because of progressive hypoxic respiratory failure. The body weight gain over 4 days was similar among three experimental groups (0.29 ± 0.02, 0.31 ± 0.02, and 0.32 ± 0.02 kg for sham-operated, saline-control and DOX, respectively). As shown in Tables 1, 2, arterial blood gases, systemic blood pressure and heart rate were monitored and recovered with no differences among all three groups over 4 days after reoxygenation. Although cardiac output, systolic and diastolic functions (dp/dt_max_, dp/dt_min_, and Tau) measured by Millar® assessment on the final day were slightly lower in the saline-control group, the differences were not statistically significant (Table 3).

**Table 3:** Left ventricular functions assessed by Millar® catheter in anesthetized newborn piglets after 4 days recovery.

	**Sham-operated**	**Saline-control**	**DOX**	***p*-value**
Heart rate (bpm)	219 ± 2	192 ± 13	215 ± 10	0.19
Cardiac output (mL/kg/min)	211 ± 17	187 ± 21	226 ± 36	0.55
Developed pressure (mmHg)	76 ± 1	96 ± 10	89 ± 6	0.19
dp/dt max (mmHg)	3,341 ± 446	3,481 ± 377	3,790 ± 343	0.73
dp/dt min (mmHg)	−4,158 ± 233	−5717 ± 728	−4,313 ± 629	0.17
Tau (ms)	17 ± 2	15 ± 1	14 ± 1	0.42

### Urine Output

The urine output for both sham-operated and DOX groups gradually increased with time, whereas the output from saline-control group remained unchanged throughout the 4-day observation period (Figure 2, Table 4). Consequently, the total urine output of saline-control group was significantly lower than that of both sham-operated and DOX groups. There was no evidence of volume overload in the animals of saline-control group.

**Table 4:** Changes in various renal biomarkers during recovery.

	**Sham-operated**	**Saline-control**	**DOX**
**URINE OUTPUT (mL/kg)**			
Day 1	27 ± 9	33 ± 7	36 ± 7
Day 2	15 ± 3	17 ± 6	30 ± 5
Day 3	52 ± 7^*^	25 ± 5	51 ± 5^*^
Day 4	61 ± 15^*^	26 ± 5	63 ± 7^*^
Total	154 ± 21^*^	101 ± 11	181 ± 17^*^
**PLASMA CREATININE (mg/dL)**			
Day 1	1.87 ± 0.31	2.18 ± 0.59	1.78 ± 0.47
Day 2	1.65 ± 0.19^*^	2.33 ± 0.34	1.45 ± 0.23^*^
Day 3	1.71 ± 0.26	1.88 ± 0.38	1.65 ± 0.21
Day 4	1.57 ± 0.41	1.94 ± 0.16	1.82 ± 0.24
**NAG/Cr RATIO (U/mg)**			
Day 1	1.96 ± 0.38^*^	3.13 ± 0.43	2.56 ± 0.34
Day 2	1.74 ± 0.28^*^	2.88 ± 0.43	2.20 ± 0.26
Day 3	1.93 ± 0.27^*^	3.00 ± 0.36	2.05 ± 0.10^*^
Day 4	1.94 ± 0.36	2.69 ± 0.26	2.18 ± 0.34
**TOTAL MMP-2 ACTIVITY (AU)**			
	0.97 ± 0.04^*^	1.32 ± 0.11	1.06 ± 0.05^*^
**TISSUE LACTATE (μmol/mg PROTEIN)**			
	0.17 ± 0.01	0.24 ± 0.04	0.19 ± 0.02

* *p < 0.05 vs. saline-control group (two-ways repeated measures or one-way ANOVA)*.

### Biochemical Parameters

**Figure 3:**
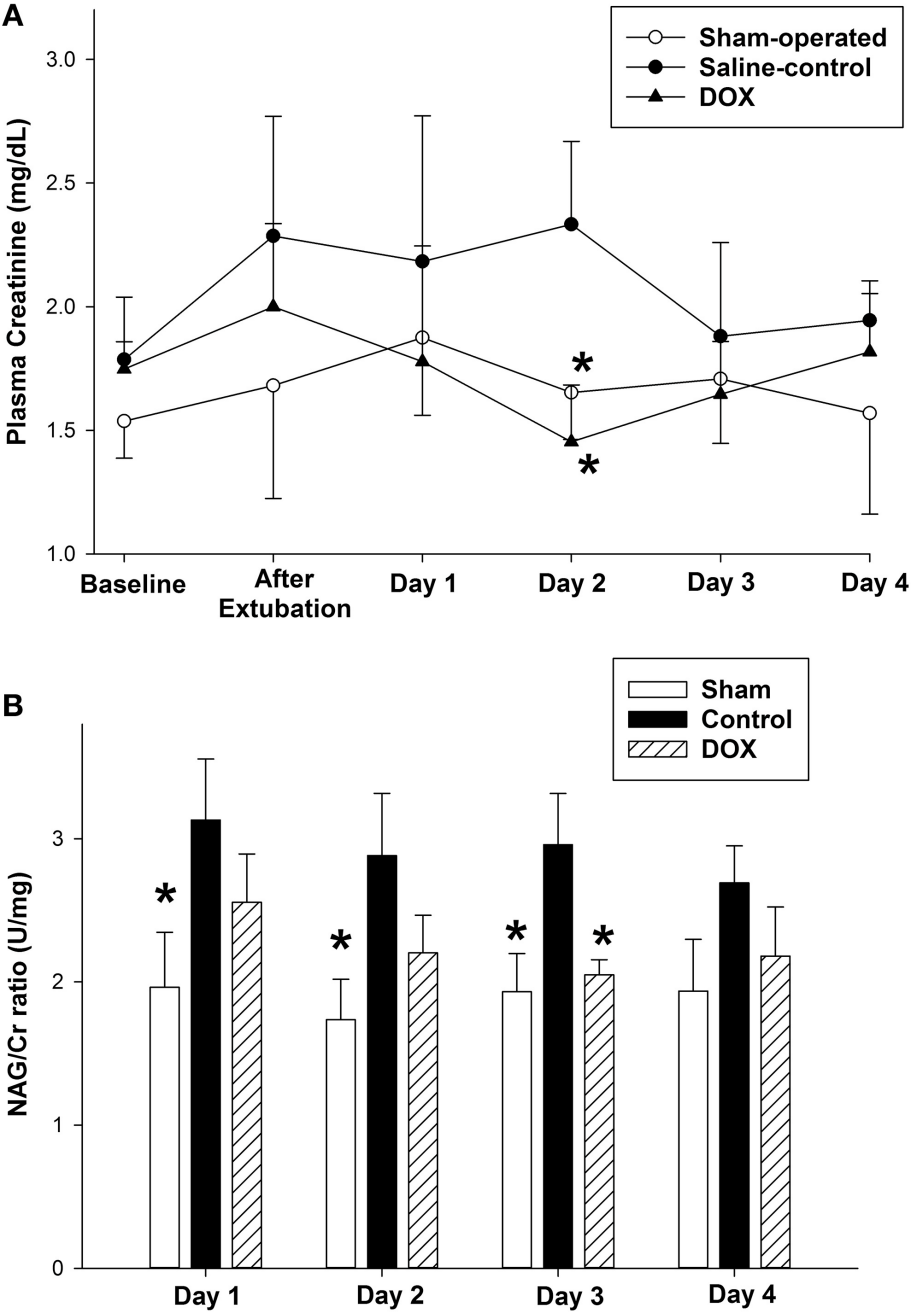
Temporal changes in **(A)** plasma creatinine level and **(B)** urine NAG/Cr ratio after hypoxia and during recovery of sham-operated, saline-control and DOX groups. All values represent mean ± SEM. ^*^p < 0.05 vs. saline-controls at current time point (two-way repeated measures ANOVA).

**Figure 4:**
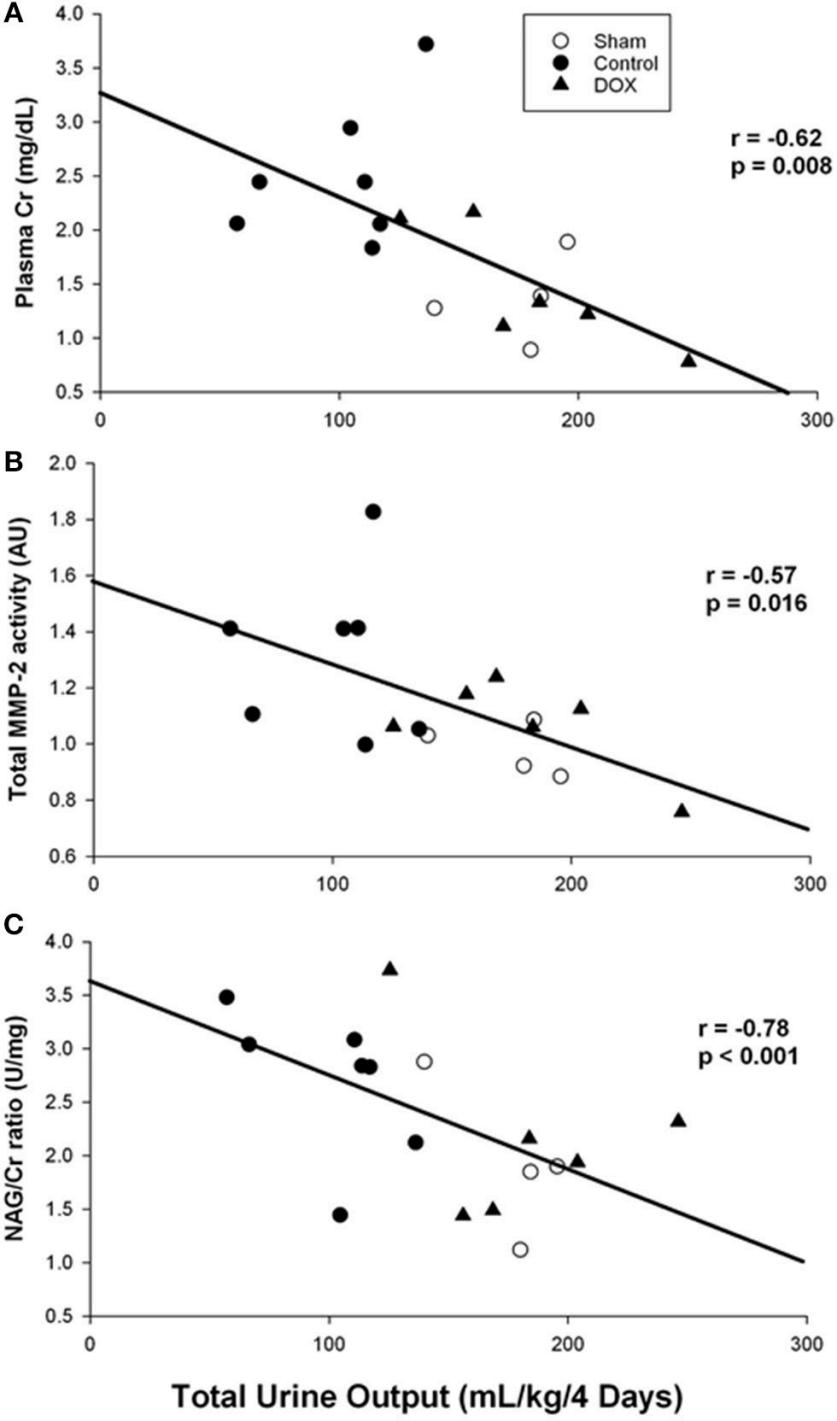
Correlation between total urine output and **(A)** plasma creatinine level, **(B)** total MMP-2 activity, and **(C)** urine NAG/Cr ratio (Pearson Product Moment test).

Table 4 summarized changes in all renal biomarkers among all experimental groups. Plasma Cr levels of both sham-operated and DOX groups increased during the experimental period, and then declined back to the baseline level during the recovery period (Figure 3A). In contrast, the plasma Cr level of saline-control group remained high during recovery and was significantly higher than both sham-operated and DOX group on day 2. The plasma Cr on day 2 was negatively correlated with total urine output (*r* = −0.62, *p* = 0.008) (Figure 4A).

**Figure 5:**
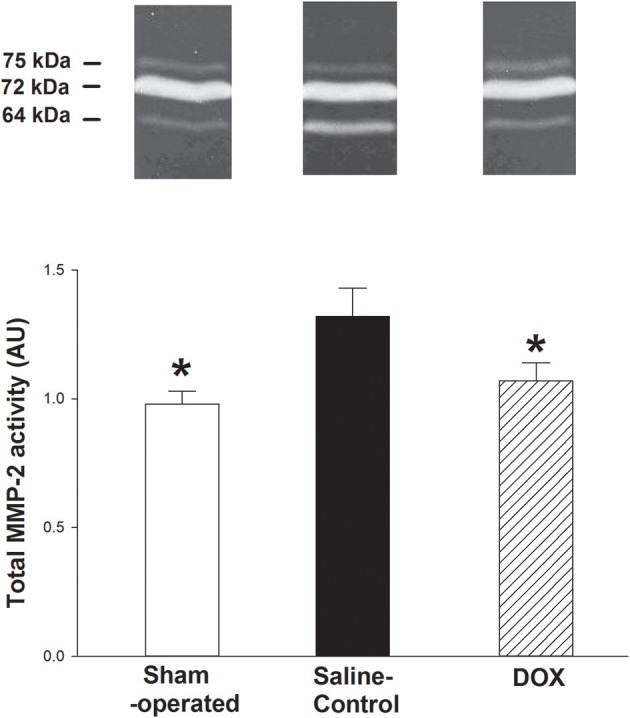
Representative zymographs of MMP-2 activity in renal tissues of hypoxic piglets after 4 days of reoxygenation, which either received saline (saline-control) or DOX. Sham-operated piglets had no hypoxia and reoxygenation. All values represent mean ± SEM. ^*^p < 0.05 vs. saline-controls (one-way ANOVA).

**Figure 6:**
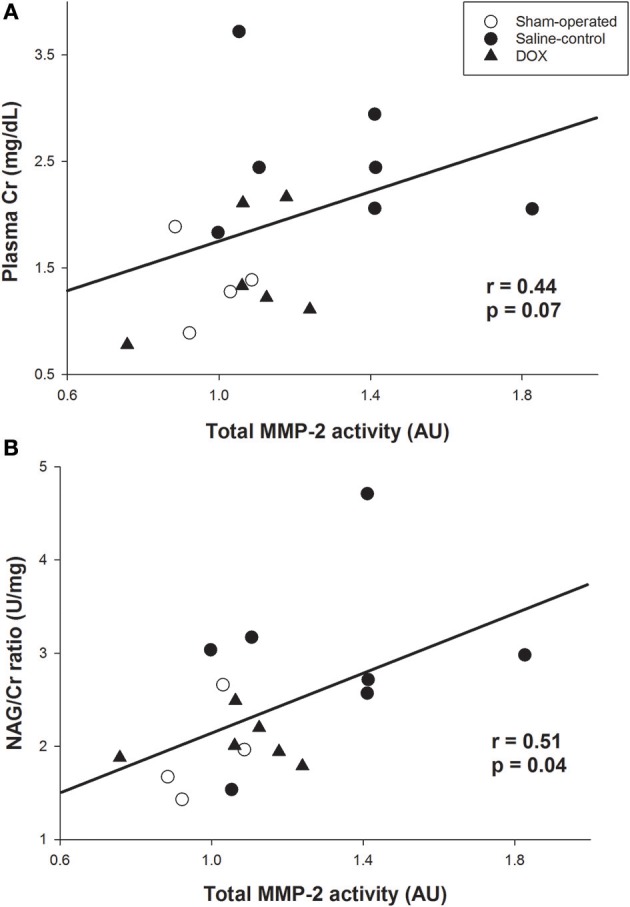
Correlation between total MMP-2 activity and **(A)** plasma creatinine level, and **(B)** urine NAG /Cr ratio (Pearson Product Moment test).

As shown in Figure 5, total MMP-2 activity of the kidney was higher in saline-control piglets. The gelatinolytic activities of MMP-2 at 75, 72, and 64 kDa were studied with significant increased activity of MMP-2 at 64 kDa of saline-control piglets, compared with sham-operated and DOX groups, but not at 75 and 72 kDa. Total MMP-2 activity had negative correlation with total urine output (*r* = −0.57, *p* = 0.016) (Figure 4B) and a positive correlation trend with plasma Cr level (*r* = 0.44, *p* = 0.07) (Figure 6A). No difference was found in MMP-9 activity among different experimental groups (data not shown).

Urine NAG/Cr ratio, an index of renal tubular function associated with H-R, of the sham-operated group remained about the same throughout the 4 days observation period (Figure 3B). The urinary NAG activities of both saline-control and DOX groups were higher than that of sham-operated group on day 1. However, the urinary NAG activity of DOX group declined with time and was significantly lower than that of saline-control group on day 3 (Figure 3B). Urinary NAG activity on day 3 was found to significantly correlate with total urine output (*r* = −0.78, *p* < 0.001) (Figure 4C) and total MMP-2 activity (*r* = −0.51, *p* = 0.04) (Figure 6B).

Four days after H-R, renal tissue lactate levels were elevated in H-R saline-control piglets (0.24 ± 0.04 μmol/mg protein) when compared with that of sham-operated and DOX piglets (0.17 ± 0.01 and 0.19 ± 0.02 μmol/mg protein, respectively), but the difference was not significant (Table 4). The renal lactate level did not correlate with total urine output (*r* = −0.41, *p* = 0.11).

## Discussion

The current study confirmed that single bolus DOX injection immediately after hypoxic insult preserved renal function for a prolonged period in surviving newborn piglets. The findings are original in an intact large animal surviving model of neonatal hypoxia and are important for the translation into clinical trials. By measuring the renal blood flow directly, we previously demonstrated a significant decrease in renal perfusion during hypoxia (18). It has also been shown that vital organs such as the heart, brain, and adrenal glands are preferentially perfused at the expense of other organ systems during hypoxia (5, 22). Reducing renal blood flow has been suggested to be the initial cause of kidney injury in hypoxic-ischemic insult. Further injury occurs during reoxygenation with the increased production of reactive oxygen and nitrogen species (23, 24). AKI in the neonatal period has been associated with oliguria/anuria and or elevated plasma Cr levels (25, 26). The daily urine output of the saline-control group remained low throughout the observation period, whereas the output of DOX group increased drastically after day 2 with a consequently higher total urine output (Figure 2). We further observed that plasma Cr level of saline-control piglets increased after H-R and remained high during the recovery period, whereas plasma Cr levels of DOX piglets gradually declined back to around the baseline level over 4 days (Figure 3A). NAG is a lysosomal enzyme present in cells of the proximal tubules. Urinary NAG activity has been suggested to be used as an indicator of AKI (26, 27), and correlated positively with the severity of asphyxia in term neonates (28). Our results demonstrated that the H-R induced AKI could be protected by DOX treatment in these newborn piglets surviving the H-R.

MMP has been shown to play a crucial role in the pathogenesis of acute ischemia/reperfusion injury of the kidney (10, 11). Our findings of increased renal MMP-2 activity and the negative correlation with urine output support this notion. By using MMP-2 deficient transgenic mouse model, the degree of acute tubular injury and renal dysfunction was markedly less in the MMP-2 deficient transgenic mice compared to that seen in the wild type mice after renal ischemia-reperfusion (12). Interestingly, we observed the increased MMP-2 activity was attenuated by DOX treatment in this hypoxia-reoxygenation injury of the kidneys. Previously, it was suggested that activated MMPs targeted cell adhesion molecules in the renal tubular cells, leading to renal tubular injury and dysfunction (29–31). This seems to be the case as there was a positive correlation between MMP-2 and NAG in our study. However, we cannot exclude other potential pathophysiologic mechanisms that may mediate AKI in H-R injury. Of note, although the use of supplemental oxygen at FiO_2_ 0.21–0.25 (which is often needed in post-surgical and anesthetic animals) might have influenced the oxidative stress and therefore MMP-2 activation, both H-R groups were treated similarly resulting in normoxic reoxygenation with FiO_2_ of 0.21–0.25 and PaO_2_ similar to baseline according to the protocol (Table 1).

In this study we also examined the left ventricular systolic and diastolic functions by the placement of a Millar® catheter in these newborn animals. We previously demonstrated that the post-resuscitation administration of doxycycline attenuated cardiac injury (plasma troponin I, myocardial lactate and MMP-2 activity) and improved functional recovery in acutely instrumented piglets with H-R (32). We therefore speculated that there might be a persistence of improved left ventricular function after 4 days, which was associated with better cardiac output and regional (renal) perfusion, in the DOX piglets. However, we did not observe any significant improvement in the cardiac function on day 4. This may be related to a transient beneficial effect in the cardiac function which resolved during the recovery, confounding effects of general anesthesia, an isolated, reno-protective of doxycycline in renal function, in addition to a small sample size in a different (acute vs. survival) neonatal H-R model. Serial echocardiographic examinations including cardiac output and superior vena cava flow measurement, doppler studies of renal artery, and the use of near-infrared spectroscopy may help answer some of these questions regarding systemic blood flow and renal perfusion.

## Limitations

Piglets have similar anatomy and physiology to humans (33). Further it has been shown that newborn piglets even experience asphyxia in a similar manner to human neonates (34, 35). Nevertheless, the translation of our findings needs extreme caution including the applicability of the model of hypoxia and asphyxia. The latter includes the absence of perinatal transition, hypercapnia and severe cardiovascular compromise in the current model despite of severe metabolic acidosis with hypoxia. Longer hypoxic challenge (120 vs. 60 min) and more invasive surgical procedures were carried out in previous acute study (18, 32) than those of current study. Further the application of therapeutic hypothermia, a standard practice for neonates with hypoxic-ischemic encephalopathy in developed countries, may confound our observations for it effects in oxidative stress-related injury.

Among all tetracyclines, DOX is approved for use in neonates and has a lesser side-effect profile (36, 37). The use of DOX is safe in children younger than 8 years, particularly for short term therapy. Nevertheless, DOX has not been widely used in neonates and has been restricted for the treatment of specific infections (13). Furthermore, it is uncertain if there were pharmacodynamic and pharmacokinetic differences in these 1–4 days-old piglets, which could be associated with the postnatal increase in renal function especially for surviving animal models. Retrospective analysis of urine output of 1–2 and 3–4 days-old piglets suggested similar trends of renal protection with the DOX treatment, compared with the saline-controls (*p* = 0.03 and *p* = 0.22, respectively). More pharmacokinetic and pharmacodynamic studies are needed for DOX in neonates. Although DOX may be the most clinically transferable MMP inhibitor for use in neonates at present, further studies on its safety in this patient population are required.

A prolonged survival experiment with investigations on the brain and intestinal microbiomes may be needed to study the safety of DOX treatment. Notwithstanding the challenges with chronic survival experiments, repetitive tissue sampling or invasive interventions will further understand the temporal changes in the mechanism of DOX including the inhibition of MMP-2 activation and other pathways such as improved mitochondrial function (38). The measurement of tissue inhibitors of MMP-2, use of *in-situ* zymography, detailed histological and electronic microscopic examinations will help study the mechanisms and location of renal injury.

## Conclusions

Using a surviving swine model of neonatal H-R, we demonstrated that a single dose of DOX given immediately after hypoxic insult improved renal function and alleviated renal injury with inhibition of MMP-2 activation in the kidneys. The reno-protective effect was weak but significant; our results provide the foundation for future studies on the long-term effects of DOX and clinical investigations of DOX in neonatal AKI after H-R.

## Data Availability

The raw data supporting the conclusions of this manuscript will be made available by the authors upon request, without undue reservation, to any qualified researcher.

## Ethics Statement

The study has been approved by the University of Alberta Animal Care and Use Committee and was conducted with full compliance to the Canadian guidelines on the use of animal for experiments.

## Author Contributions

GS and P-YC: conception and design; T-FL, ML, MP, GS, and P-YC: collection and assembly of data, analysis, and interpretation of the data; T-FL, GS, and P-YC: drafting of the article; T-FL, ML, MP, GS, and P-YC: critical revision of the article for important intellectual content and final approval.

### Conflict of Interest Statement

The authors declare that the research was conducted in the absence of any commercial or financial relationships that could be construed as a potential conflict of interest.

## References

[1] Lawn JE. Estimating the causes of 4 million neonatal deaths in the year 2000. Int J Epidemiol. (2006) 35:706–18. 10.1093/ije/dyl043 16556647

[2] Liu L Johnson HL Cousens S Perin J Scott S Lawn JE. Global, regional, and national causes of child mortality: an updated systematic analysis for 2010 with time trends since 2000. Lancet. (2012) 379:2151–61. 10.1016/S0140-6736(12)60560-1 22579125

[3] Shah P. Multiorgan dysfunction in infants with post-asphyxial hypoxicischaemic encephalopathy. Arch Dis Child Fetal Neonatal Ed. (2004) 89: F152–5. 10.1136/adc.2002.023093 14977901 PMC1756028

[4] Sheira G Noreldin N Tamer A Saad M. Urinary biomarker N-acetyl-β-D-glucosaminidase can predict severity of renal damage in diabetic nephropathy. J Diabetes Metab Disord. (2015) 14:4. 10.1186/s40200-015-0133-6 25717442 PMC4340101

[5] Aggarwal A Kumar P Chowdhary G Majumdar S Narang A. Evaluation of renal functions in asphyxiated newborns. J Trop Pediatr. (2005) 51:295–9. 10.1093/tropej/fmi017 16000344

[6] Durkan AM Alexander RT. Acute kidney injury post neonatal asphyxia. J Pediatr. (2011) 158:e29–33. 10.1016/j.jpeds.2010.11.010 21238703

[7] Nankervis CA Giannone PJ Reber KM. The neonatal intestinal vasculature: contributing factors to necrotizing enterocolitis. Semin Perinatol. (2008) 32:83–91. 10.1053/j.semperi.2008.01.003 18346531

[8] Perlman JM Tack ED. Renal injury in the asphyxiated newborn infant: relationship to neurologic outcome. J Pediatr. (1988) 113:875–9. 10.1016/S0022-3476(88)80023-4 3054034

[9] Kandasamy AD Chow AK Ali MAM Schulz R. Matrix metalloproteinase−2 and myocardial oxidative stress injury: beyond the matrix. Cardiovasc Res. (2010) 85:413–23. 10.1093/cvr/cvp268 19656780

[10] Han WK Walkar SS Johnson A Betensky RA Dent CL Devarajan P. Urinary biomarkers in the early diagnosis of acute kidney injury. Kidney Int. (2008) 73:863–9. 10.1038/sj.ki.5002715 18059454 PMC2586909

[11] Zhao H Dong Y Tian X Tan TK Liu Z Zhao Y. Matrix metalloproteinases contribute to kidney fibrosis in chronic kidney diseases. World J Nephrol. (2013) 2:84–9. 10.5527/wjn.v2.i3.84 24255890 PMC3832915

[12] Kunugi S Shimizu A Kuwahara N Du X Takahashi M Terasaki Y. Inhibition of matrix metalloproteinases reduces ischemia-reperfusion acute kidney injury. Lab Invest. (2011) 91:170–80. 10.1038/labinvest.2010.174 20956976

[13] Seese RT Danziger-Isakov L Chapter 325: Rocky Mountain spotted fever. In: Textbook of Pediatric Care, 2nd ed. Elk Grove Village, IL: American Academy of Pediatrics (2012).

[14] Griffin MO Fricovsky E Ceballos G Villarreal F. Tetracyclines: a pleitropic family of compounds with promising therapeutic properties. Review of the literature. Am J Physiol Cell Physiol. (2010) 299:C539–48. 10.1152/ajpcell.00047.2010 20592239 PMC2944325

[15] Sapadin AN Fleischmajer R. Tetracyclines: nonantibiotic properties and their clinical implications. J Am Acad Dermatol. (2006) 54:258–65. 10.1016/j.jaad.2005.10.004 16443056

[16] Ihtiyar E Yazar NF Erkasap N Koken T Tosun M Oner S. Effects of doxycycline on renal ischemia reperfusion injury induced by abdominal compartment syndrome. J Surg Res. (2011) 167:113–20. 10.1016/j.jss.2009.09.048 20080260

[17] Kucuk A Kabadere S Tosun M Koken T Kinaci MK Isikli B. Protective effects of doxycycline in ischemia/reperfusion injury on kidney. J Physiol Biochem. (2009) 65:183–91. 10.1007/BF03179069 19886397

[18] LaBossiere JR Pelletier JS Thiesen A Schulz R Bigam DL Cheung PY. Doxycycline attenuates renal injury in a swine model of neonatal hypoxia-reoxygenation. Shock. (2015) 43:99–105. 10.1097/SHK.0000000000000257 25105465

[19] Kilkenny C Browne WJ Cuthill IC Emerson M Altman DG. Improving bioscience research reporting. The ARRIVE guidelines for reporting animal research. PLoS Biol. (2010) 8:e1000412–5. 10.1371/journal.pbio.1000412 20613859 PMC2893951

[20] Wykes LJ Ball RO Pencharz PB. Development and validation of a total parenteral nutrition model in the neonatal piglet. J Nutr. (1993) 123:1248–59. 10.1093/jn/123.7.1248 8320564

[21] Haase E Bigam DL Nakonechny QB Rayner D Korbutt G Cheung PY Cardiac function, myocardial glutathione, and matrix metalloproteinase−2 levels in hypoxic newborn pigs reoxygenated by 21%, 50%, or 100% oxygen. Shock. (2005) 23:383–9. 10.1097/01.shk.0000158962.83529.ce 15803064

[22] Kuwahira I Gonzalez NC Heisler N Piiper J. Changes in regional blood flow distribution and oxygen supply during hypoxia in conscious rats. J Appl Physiol. (1993) 74:211–4. 10.1152/jappl.1993.74.1.211 8444693

[23] Bonventre JV Yang L. Cellular pathophysiology of ischemic acute kidney injury. J Clin Invest. (2011) 121:4210–21. 10.1172/JCI45161 22045571 PMC3204829

[24] Fink MP. Reactive oxygen species as mediators of organ dysfunction caused by sepsis, acute respiratory distress syndrome, or hemorrhagic shock: potential benefits of resuscitation with Ringer's ethyl pyruvate solution. Curr Opin Clin Nutr Metab Care. (2002) 5:167–74. 11844984 10.1097/00075197-200203000-00009

[25] Askenazi D. Are we ready for the clinical use of novel acute kidney injury biomarkers? Pediatr Nephrol. (2012) 27:1423–1425. 10.1007/s00467-012-2185-x 22689085

[26] Singh KS Sengar GS A study of multiorgan dysfunction in asphyxiated neonates. Int J Contemp Pediatr. (2016) 3:625–30. 10.18203/2349-3291.ijcp20161052

[27] Kovarikova S Urinary biomarkers of renal function in dogs and cats: a review. Vet Med. (2015) 60:589–602. 10.17221/8527-VETMED

[28] Vento M Sastre J Asensi MA Viña J. Room-air resuscitation causes less damage to heart and kidney than 100% oxygen. Am J Respir Crit Care Med. (2005) 172:1393–8. 10.1164/rccm.200412-1740OC 16141440

[29] Catania JM Chen G Parrish AR. Role of matrix metalloproteinases in renal pathophysiologies. Am J Physiol Renal Physiol. (2006) 292:F905–911. 10.1152/ajprenal.00421.2006 17190907

[30] Cortes AL Gonsalez SR Rioja LS Oliveira SSC Santos ALS Prieto MC. Protective outcomes of low-dose doxycycline on renal function of Wistar rats subjected to acute ischemia/reperfusion injury. Biochim Biophys Acta. (2018) 1864:102–14. 10.1016/j.bbadis.2017.10.005 28987762 PMC5705293

[31] Covington MD. Ischemia-induced cleavage of cadherins in NRK cells: evidence for a role of metalloproteinases. Am J Physiol Renal Physiol. (2005) 289:F280–8. 10.1152/ajprenal.00351.2004 15769936

[32] LaBossiere JR Pelletier JS Ali MAM Thiesen A Schulz R Bigam DL. Postresuscitation administration of doxycycline preserves cardiac function in hypoxia-reoxygenation injury of newborn piglets. Crit Care Med. (2014) 42:e260–9. 10.1097/CCM.0000000000000135 24335448

[33] Swindle M Comparative anatomy and physiology of the pig. Scand J Lab Anim Sci. (1998) 25:11–21.

[34] Alonsospilsbury M Motarojas D Villanuevagarcia D Martinezburnes J Orozco H Ramireznecoechea R Perinatal asphyxia pathophysiology in pig and human: a review. Anim Reprod Sci. (2005) 90:1–30. 10.1016/j.anireprosci.2005.01.007 16257594

[35] Chapados I Cheung PY. Not all models are created equal: animal models to study hypoxic-ischemic encephalopathy of the newborn. Neonatology. (2008) 94:300–3. 10.1159/000151650 18784427

[36] Heaton PC Fenwick SR Brewer DE. Association between tetracycline or doxycycline and hepatotoxicity: a population based case-control study. J Clin Pharm Ther. (2007) 32:483–7. 10.1111/j.1365-2710.2007.00853.x 17875115

[37] Sloan B Scheinfeld N. The use and safety of doxycycline hyclate and other second-generation tetracyclines. Expert Opin Drug Saf. (2008) 7:571–7. 10.1517/14740338.7.5.571 18759709

[38] Moser MA Arcand S Lin HB Wojnarowicz C Sawicka J Banerjee T. Protection of the transplant kidney from preservation injury by inhibition of matrix metalloproteinases. PLoS One. (2016) 11:e0157508. 10.1371/journal.pone.0157508 27327879 PMC4915675

